# Supplementary material for: Influence of Estrogen Treatment on ESR1+ and ESR1− Cells in ER+ Breast Cancer: Insights from Single-Cell Analysis of Patient-Derived Xenograft Models
Source: Cancers (Basel). 2021 Dec 19;13(24):6375. doi: 10.3390/cancers13246375 (PMC8699443; doi:10.3390/cancers13246375)
Supplement: Supplementary file 1 [file cancers-13-06375-s001.zip › cancers-1493701-Supp-Proofreading.pdf]

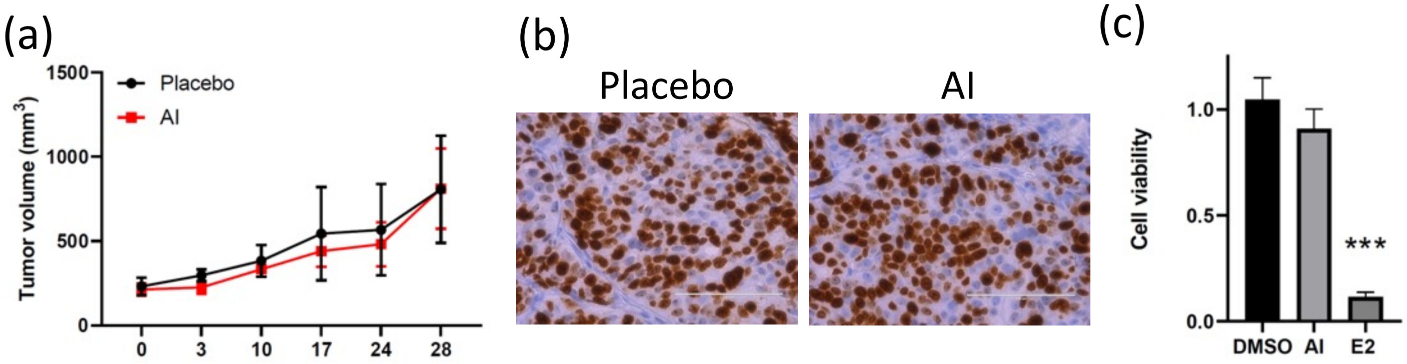

Figure S1 Aromatase inhibitor (AI)/placebo treatments to GS3-PDX: (**a**) Tumor growth curve of GS3-PDX with AI (letrozole, 10 mg, daily s.c.) or placebo treatment for 28 days; (**b**) immunohistochemistry of Ki-67 expression in GS3 tumors treated with AI or placebo; (**c**) Cell viability of 10^4 organoids (isolated from GS3 tumor) treated with DMSO (Dimethyl sulfoxide), AI (letrozole, 200nM), or E2 (10nM) for 96 hours *in vitro* (***p < 0.0001); Error bars represent SEM.

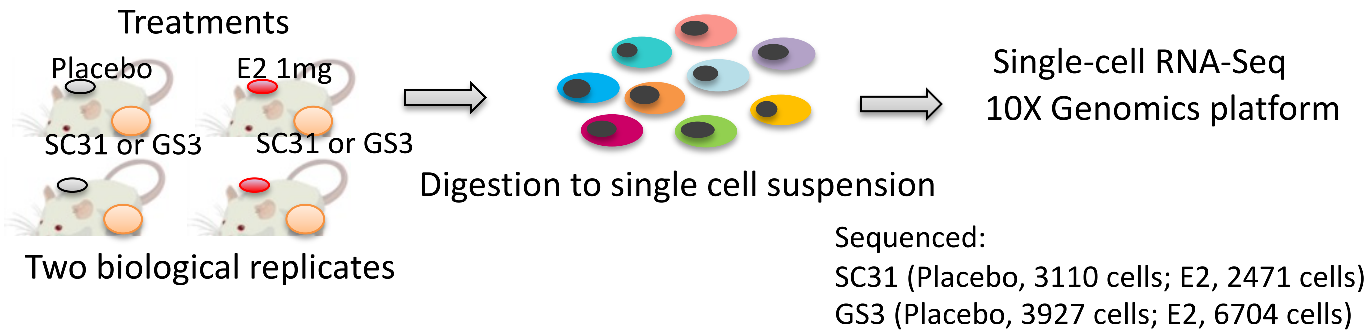

Figure S2 Overview of scRNA-Seq approach using two PDX models.

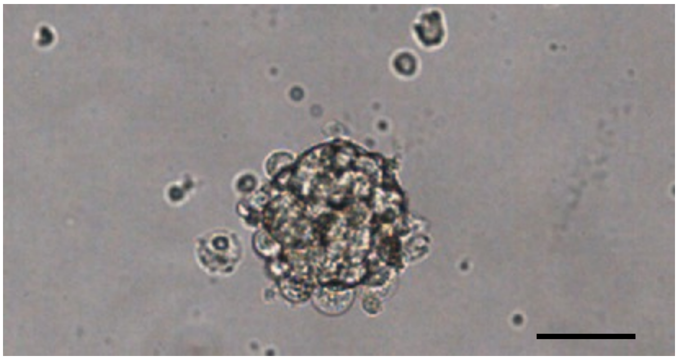

Figure S3 Organoids established from GS3-PDX tumors. Organoids were established from single cells isolated from fresh GS3 tumor. This organoid is control sample (Dimethyl sulfoxide) incubated for 3 days. Scale bar represents 50 μm.

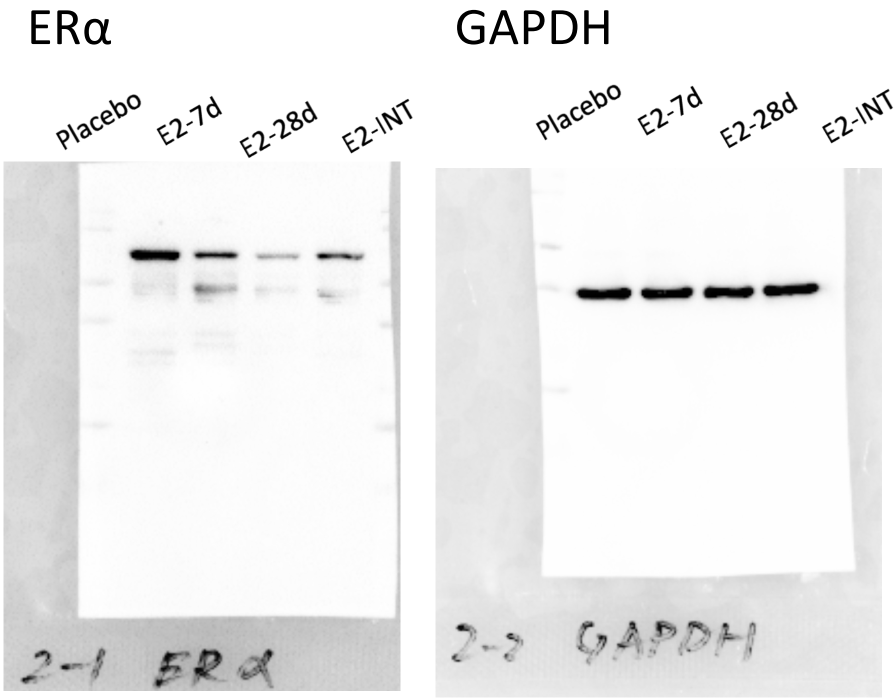

Figure S4 Whole blots of ER*α* expression of GS3 tumors treated with placebo, 7-day E2, 28-day E2 and intermittent E2 by Western blotting.

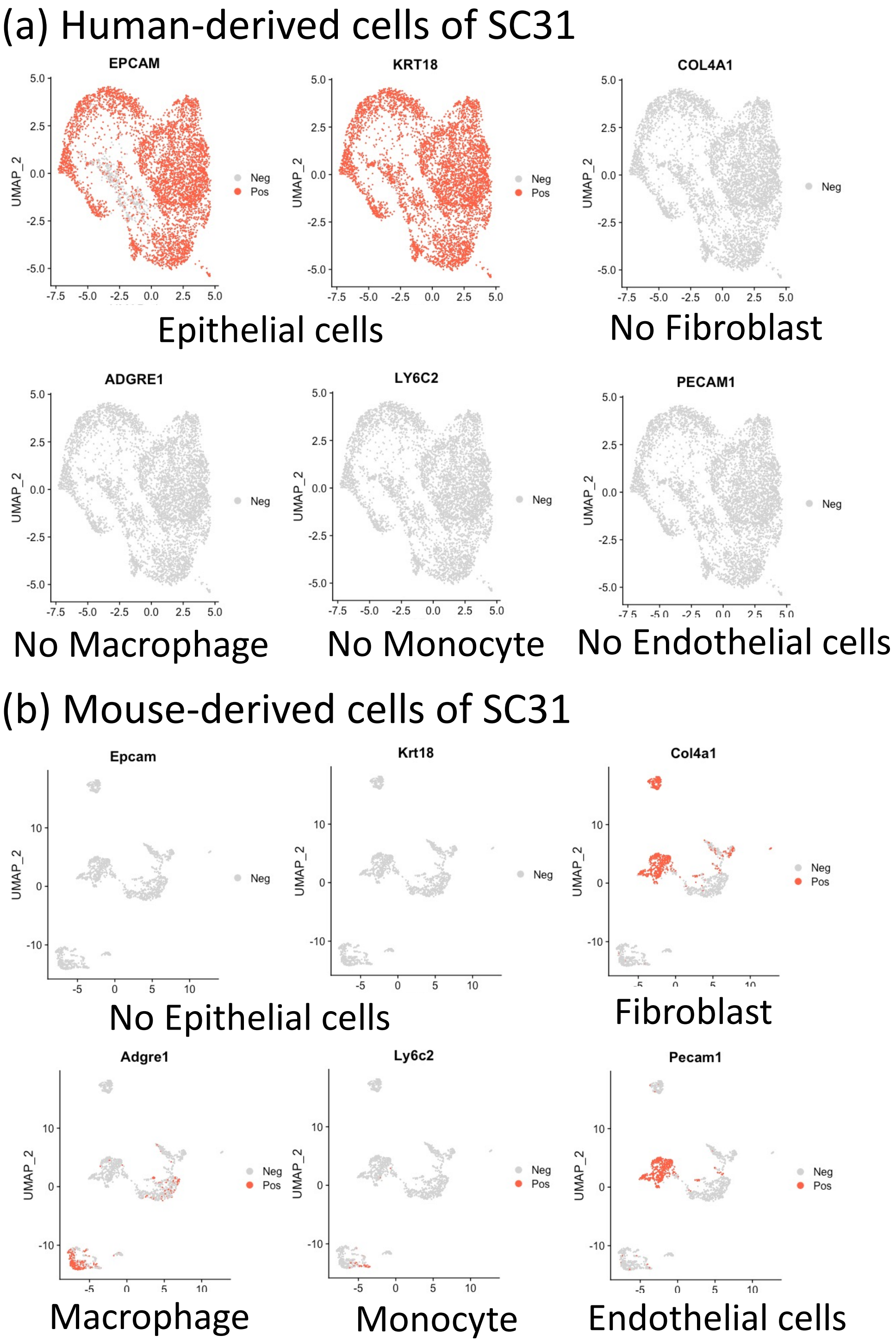

Figure S5 (1/2) Epithelial and stromal cell markers in human/mouse-derived cells in SC31 (a, b) and GS3 (c, d).

(c) Human-derived cells of GS3

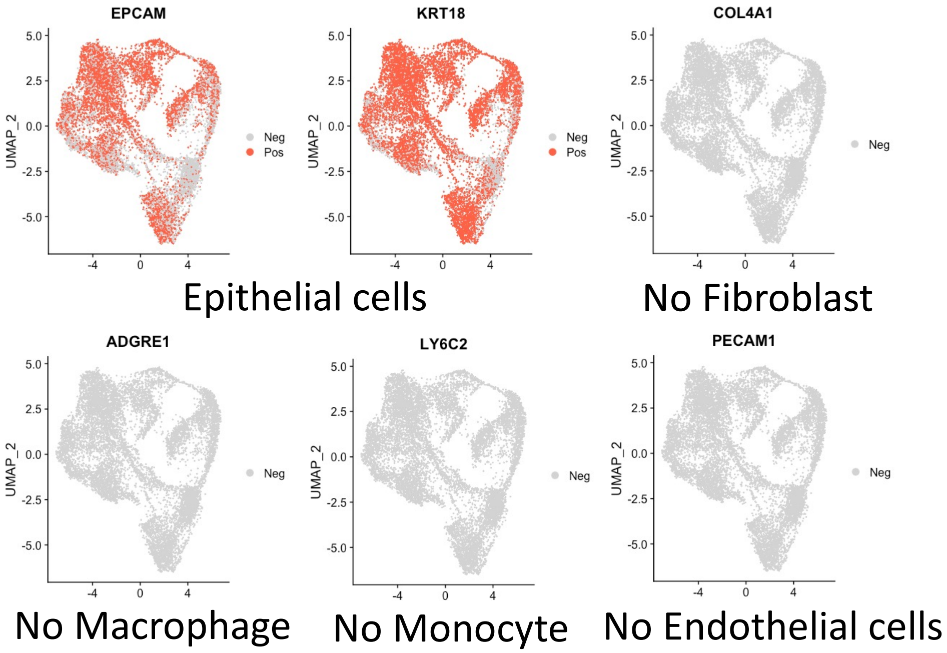

(d) Mouse-derived cells of GS3

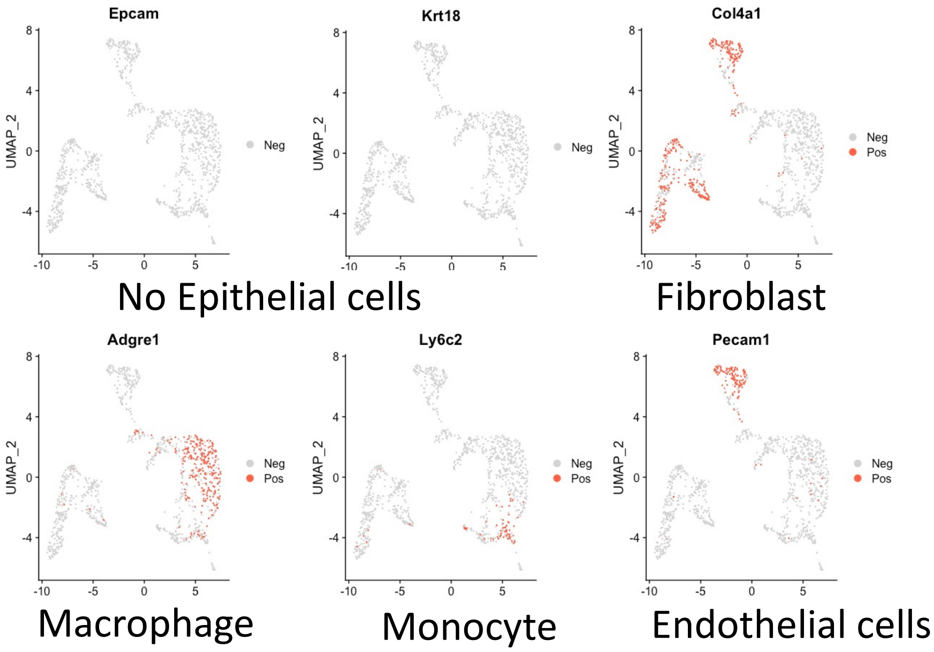

Figure S5 (2/2) Epithelial and stromal cell markers in human/mouse-derived cells in SC31 (a, b) and GS3 (c, d).

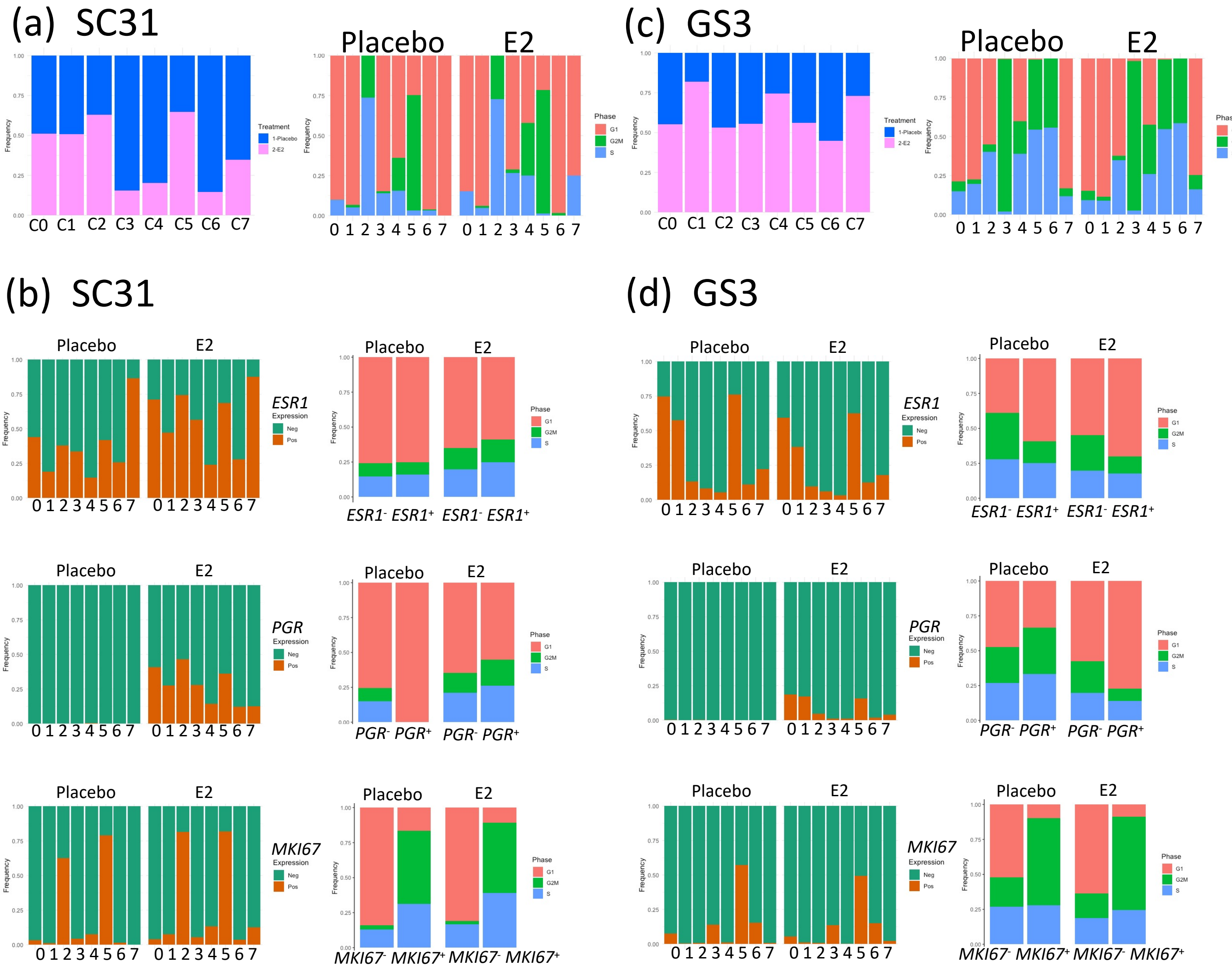

Figure S6 Cell distribution of single-cell clusters by treatments and by cell cycle phases (separated by treatments) in (**a**) SC31 and (**c**) GS3; Cell distribution of single cell clusters and cell cycle phases by gene expression (separated by treatments) in (**b**) SC31 and (**d**) GS3.

(a) SC31

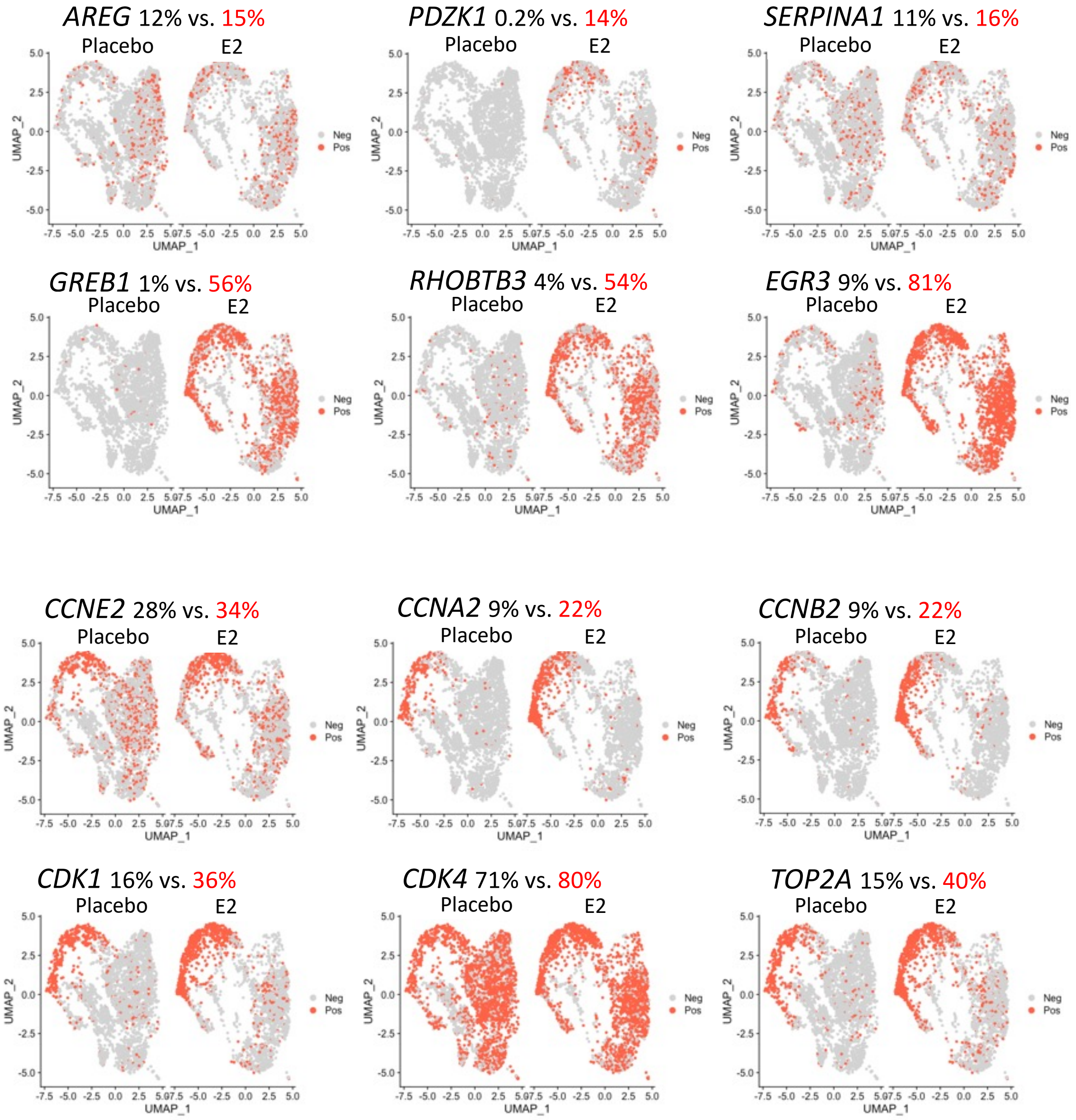

Figure S7 (1/2) Gene expression (estrogen-regulated genes and cell cycle proliferation genes) comparison of E2-treated cells versus placebo-treated cells in (**a**) SC31 and (**b**) GS3.

(b) GS3

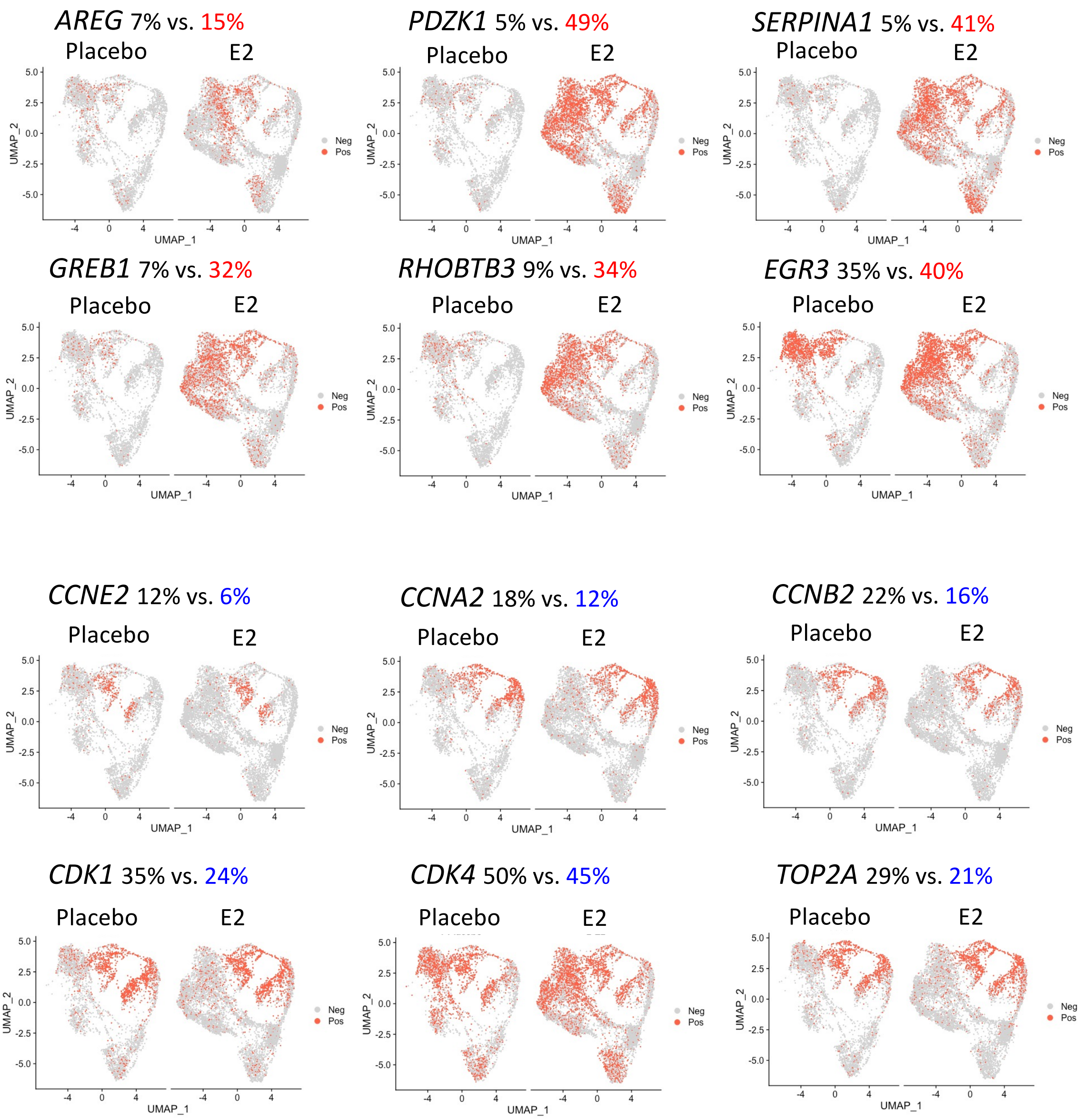

Figure S7 (2/2) Gene expression (estrogen-regulated genes and cell cycle proliferation genes) comparison of E2-treated cells versus placebo-treated cells in (**a**) SC31 and (**b**) GS3.

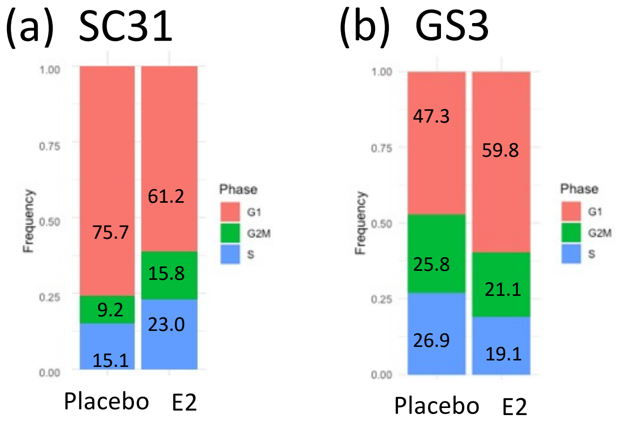

Figure S8 Distribution of cells in the G1, S, or G2M phases separated by treatments in (**a**) SC31 and (**b**) GS3.

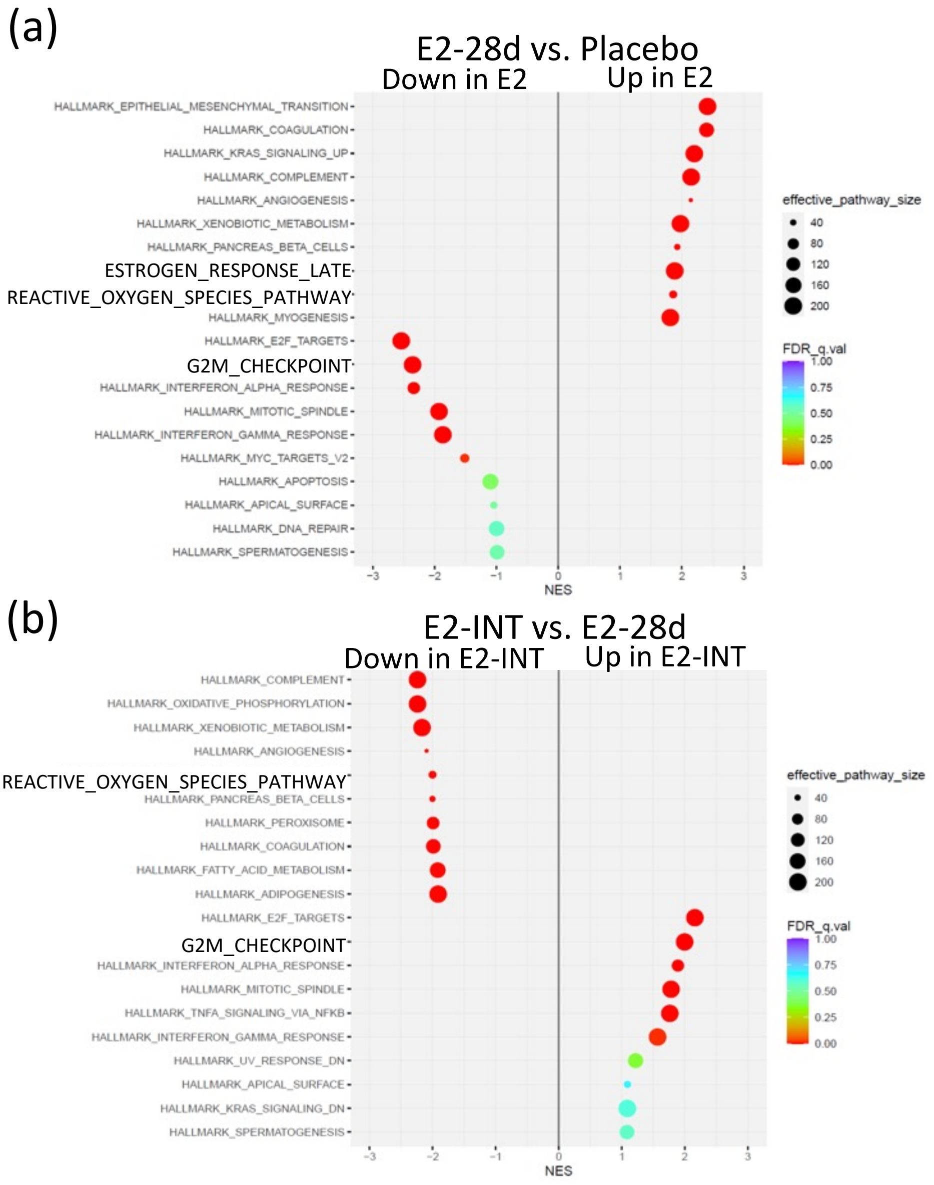

Figure S9 Analysis of hallmark gene sets based on bulk RNA-Seq data of intermittent E2 sample in GS3: (**a**) Analysis of hallmark gene sets of 28-day E2 treatment versus placebo; (**b**) Analysis of hallmark gene sets of intermittent E2 versus 28-day E2 treatment; E2-28d, E2 treatment for 28 days; E2-INT, intermittent E2 treatment.

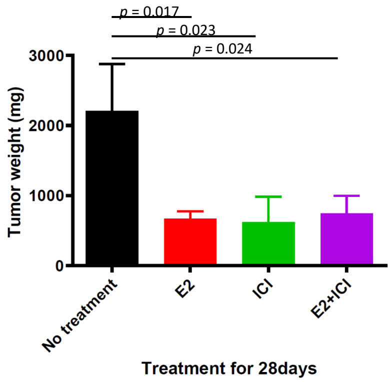

Figure S10 Tumor weight of GS3 after 4 weeks of placebo/E2/ICI/E2+ICI treatment; Error bars represent SEM and *p* value was determined by t-test.

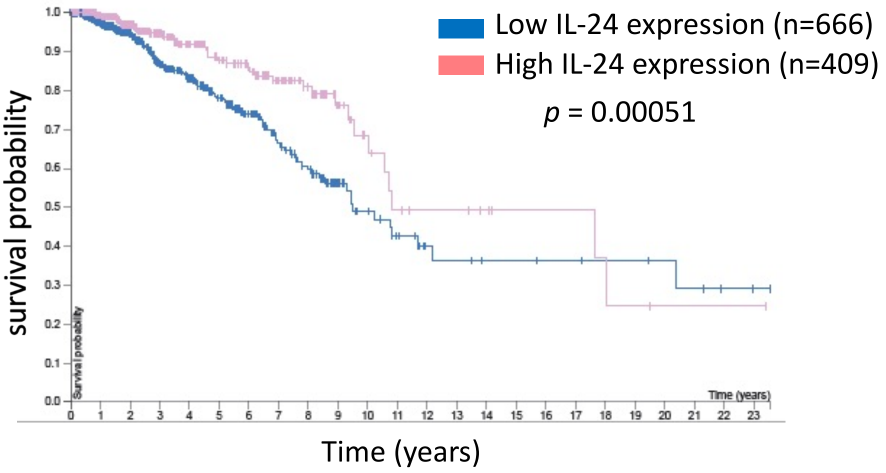

Figure S11 Prognostic value of IL-24 expression: Kaplan-Meier curves showing estimating overall survival for IL-24 expression according to the human protein atlas (https://www.proteinatlas.org/ENSG00000162892-IL24/pathology/breast+cancer, Accessed on 11/18/2021); *p* value is for comparison of two groups.

Table S1 Primer sequences

Genes		Sequence (5′->3′)	Tm (℃)	Amplicon Size (bp)
ESR1	Forward	AAG CTT CGA TGA TGG GCT TA	54.0	145
	Reverse	AGG ATC TCT AGC CAG GCA CA	57.7	
ESR2	Forward	CCA TGA TCC TGC TCA ATT CC	53.5	150
	Reverse	ATT GCT GCT GGG AGG AGA T	56.3	
PGR	Forward	CTT AAT CAA CTA GGC GAG AG	49.9	122
	Reverse	AAG CTC ATC CAA GAA TAC TG	49.6	
IL24	Forward	CTT TGT TCT CAT CGT GTC ACA AC	54.3	114
	Reverse	TCC AAC TGT TTG AAT GCT CTC C	55.3	
GADD45A	Forward	GGT GTA CGA AGC GGC CAA	58.3	59
	Reverse	GCA GGC ACA ACA CCA CGT TA	58.4	
GREB1	Forward	CAA AGA ATA ACC TGT TGG CCC TGC	58.7	172
	Reverse	GAC ATG CCT GCG CTC TCA TAC TTA	58.8	
TFF1	Forward	GTG TCA CGC CCT CCC AGT	60.2	63
	Reverse	GGA CCC CAC GAA CGG TG	58.9	
ERBB2	Forward	AAA GGC CCA AGA CTC TCT CC	56.6	89
	Reverse	CAA GTA CTC GGG GTT CTC CA	56.2	
ACTB	Forward	CAA CTG GGA CGA CAT GGA GA	56.7	170
	Reverse	ACG TAC ATG GTG GGG TGT TG	57.6	
GAPDH	Forward	GGT CTC CTC TGA CTT CAA CA	53.8	116
	Reverse	AGC CAA ATT CGT TGT CAT AC	50.9	

Table S2 Analysis of hallmark gene sets based on bulk RNA-Seq data of different treatment samples in GS3

Treatment	Upregulated in E2 (vs. control)	*p*-value	Downregulated in E2 (vs. control)	*p*-value
E2-5 days	ESTROGEN_RESPONSE_EARLY	1.43×10^{-31}	P53_PATHWAY	2.93×10^{-06}
	ESTROGEN_RESPONSE_LATE	2.57×10^{-29}	MYOGENESIS	9.75×10^{-05}
	TNFA_SIGNALING_VIA_NFKB	6.51×10^{-09}	KRAS_SIGNALING_DN	4.57×10^{-04}
E2-10 days	ESTROGEN_RESPONSE_EARLY	3.03×10^{-27}	G2M_CHECKPOINT	1.21×10^{-24}
	ESTROGEN_RESPONSE_LATE	4.60×10^{-23}	MITOTIC_SPINDLE	3.59×10^{-12}
	PANCREAS_BETA_CELLS	4.94×10^{-11}	E2F_TARGETS	3.87×10^{-11}

Footnote: This table included top three hallmark gene sets.

Table S3 Human-derived cells and mouse-derived cells in SC31 and GS3

	**SC31**	**GS3**
Human-placebo	3,110	3,927
Human-E2	2,471	6,704
Human	5,581	10,631
Mouse-placebo	1,143	224
Mouse-E2	356	905
Mouse	1,499	1,129

Table S4 The number of cells in each cluster of single-cells in SC31 and GS3

**SC31**	C0	C1	C2	C3	C4	C5	C6	C7	Total
Placebo	1,050	351	263	496	445	157	333	15	3,110
E2	1,102	364	448	90	112	290	57	8	2,471
Total	2,152	715	711	586	557	447	390	23	5,581
**GS3**	C0	C1	C2	C3	C4	C5	C6	C7	total
Placebo	1,163	409	690	527	277	392	315	154	3,927
E2	1,427	1,847	784	662	809	500	257	418	6,704
Total	2,590	2,256	1,474	1,189	1,086	892	572	572	10,631

Table S5 Analysis of GSEA hallmark gene sets based on scRNA-Seq data in each cluster of SC31

Cluster	Upregulated Gene Set Name	*p*-value	Downegulated Gene Set Name	*p*-value
C0	TNFA_SIGNALING_VIA_NFKB	9.08×10^{-45}	E2F_TARGETS	2.15×10^{-73}
	ESTROGEN_RESPONSE_LATE	1.92×10^{-13}	G2M_CHECKPOINT	7.77×10^{-54}
	ESTROGEN_RESPONSE_EARLY	4.33×10^{-12}	HYPOXIA	4.86×10^{-23}
	HYPOXIA	4.33×10^{-12}		
C1	HYPOXIA	4.24×10^{-62}	E2F_TARGETS	2.92×10^{-55}
	GLYCOLYSIS	2.94×10^{-34}	MTORC1_SIGNALING	8.01×10^{-42}
	MTORC1_SIGNALING	1.19×10^{-17}	MYC_TARGETS_V1	8.01×10^{-42}
C2	E2F_TARGETS	6.72×10^{-208}	P53_PATHWAY	3.55×10^{-14}
	G2M_CHECKPOINT	1.73×10^{-111}	INTERFERON_GAMMA_RESPONSE	3.66×10^{-13}
	MYC_TARGETS_V1	1.38×10^{-79}	APOPTOSIS	8.21×10^{-13}
C3	INTERFERON_ALPHA_RESPONSE	2.76×10^{-84}	TNFA_SIGNALING_VIA_NFKB	1.31×10^{-71}
	INTERFERON_GAMMA_RESPONSE	4.29×10^{-76}	E2F_TARGETS	3.41×10^{-51}
	COMPLEMENT	6.25×10^{-13}	HYPOXIA	4.65×10^{-41}
C4	G2M_CHECKPOINT	2.95×10^{-10}	TNFA_SIGNALING_VIA_NFKB	2.07×10^{-20}
	P53_PATHWAY	2.95×10^{-10}	ESTROGEN_RESPONSE_LATE	3.58×10^{-18}
	MITOTIC_SPINDLE	2.05×10^{-09}	MTORC1_SIGNALING	4.39×10^{-17}
C5	G2M_CHECKPOINT	2.72×10^{-112}	INTERFERON_GAMMA_RESPONSE	5.42×10^{-29}
	E2F_TARGETS	7.16×10^{-98}	INTERFERON_ALPHA_RESPONSE	2.41×10^{-25}
	MITOTIC_SPINDLE	1.00×10^{-42}	HYPOXIA	1.31×10^{-15}
C6	MTORC1_SIGNALING	3.30×10^{-41}	E2F_TARGETS	1.33×10^{-70}
	APOPTOSIS	1.40×10^{-25}	G2M_CHECKPOINT	1.36×10^{-40}
	UNFOLDED_PROTEIN_RESPONSE	3.94×10^{-25}	ESTROGEN_RESPONSE_LATE	2.35×10^{-39}
C7	OXIDATIVE_PHOSPHORYLATION	8.89×10^{-52}	ESTROGEN_RESPONSE_LATE	6.09×10^{-28}
	ADIPOGENESIS	3.81×10^{-11}	ESTROGEN_RESPONSE_EARLY	6.65×10^{-24}
	HEME_METABOLISM	3.81×10^{-11}	TNFA_SIGNALING_VIA_NFKB	3.17×10^{-15}

Footnote: This table included top three hallmark gene sets.

Table S6 Analysis of GSEA hallmark gene sets based on scRNA-Seq data in each cluster of GS3

Cluster	Upregulated Gene Set Name	*p*-value	Downegulated Gene Set Name	*p*-value
C0	TNFA_SIGNALING_VIA_NFKB	1.33×10^{-53}	MYC_TARGETS_V1	7.44×10^{-36}
	APOPTOSIS	1.09×10^{-15}	E2F_TARGETS	6.13×10^{-29}
	HYPOXIA	5.09×10^{-13}	G2M_CHECKPOINT	2.02×10^{-22}
C1	ESTROGEN_RESPONSE_LATE	3.90×10^{-07}	MYC_TARGETS_V1	5.13×10^{-65}
	ESTROGEN_RESPONSE_EARLY	5.13×10^{-06}	E2F_TARGETS	1.19×10^{-37}
	PROTEIN_SECRETION	1.81×10^{-05}	G2M_CHECKPOINT	5.62×10^{-25}
C2	OXIDATIVE_PHOSPHORYLATION	7.48×10^{-33}	G2M_CHECKPOINT	3.76×10^{-25}
	MYC_TARGETS_V1	3.45×10^{-13}	E2F_TARGETS	1.03×10^{-23}
	ADIPOGENESIS	5.50×10^{-12}	TNFA_SIGNALING_VIA_NFKB	9.61×10^{-11}
C3	MYC_TARGETS_V1	3.52×10^{-66}	TNFA_SIGNALING_VIA_NFKB	2.12×10^{-35}
	G2M_CHECKPOINT	2.76×10^{-59}	ESTROGEN_RESPONSE_EARLY	4.71×10^{-17}
	E2F_TARGETS	2.97×10^{-54}	ESTROGEN_RESPONSE_LATE	1.40×10^{-14}
C4	REACTIVE_OXYGEN_SPECIES_PATHWAY	2.99×10^{-05}	TNFA_SIGNALING_VIA_NFKB	6.12×10^{-07}
	ESTROGEN_RESPONSE_EARLY	1.05×10^{-04}	E2F_TARGETS	2.46×10^{-04}
	HYPOXIA	1.05×10^{-04}	G2M_CHECKPOINT	2.46×10^{-04}
C5	E2F_TARGETS	9.16×10^{-80}	MTORC1_SIGNALING	1.62×10^{-08}
	G2M_CHECKPOINT	1.77×10^{-48}	P53_PATHWAY	1.06×10^{-05}
	MYC_TARGETS_V1	1.80×10^{-13}	UNFOLDED_PROTEIN_RESPONSE	2.21×10^{-05}
C6	MYC_TARGETS_V1	2.46×10^{-97}	TNFA_SIGNALING_VIA_NFKB	8.16×10^{-31}
	E2F_TARGETS	3.78×10^{-78}	ESTROGEN_RESPONSE_EARLY	7.28×10^{-20}
	G2M_CHECKPOINT	5.57×10^{-41}	APOPTOSIS	4.36×10^{-14}
C7	MYC_TARGETS_V1	6.62×10^{-13}	OXIDATIVE_PHOSPHORYLATION	4.05×10^{-22}
	UNFOLDED_PROTEIN_RESPONSE	1.89×10^{-11}	E2F_TARGETS	8.10×10^{-21}
	P53_PATHWAY	1.30×10^{-09}	ESTROGEN_RESPONSE_LATE	4.71×10^{-17}

Footnote: This table included top three hallmark gene sets.

Table S7 The percentage of *ESR1*$^{+}$ and *ESR1*$^{-}$ cells in two tumors treated with placebo or E2

	ESR1^{+} cells		***ESR1***$^{-}$ cells	
	Placebo	E2	Placebo	E2
**SC31**	33%	64%	67%	36%
**GS3**	41%	31%	59%	69%

Table S8 Reported E2 therapies for AI resistant ER^+ breast cancer

Reference	Treatments	Objective Response Rate (CR+PR) (%)	Clinical Benefit Rate (CR+PR+SD) (%)
Lønning et al, 2001	DES: 5 mg, three times a day	10/32 (31)	12/32 (38)
Agrawal et al, 2006	EE: 1 mg	3/12 (25)	4/12 (33%)
Ellis et al, 2009	E2: 6 mg vs. 30 mg	6 mg, 3/34 (9)<br>30 mg,1/32 (3)	6 mg, 10/34 (29)<br>30 mg, 9/32 (28)
Mahtani et al, 2009	DES: 5 mg, three times a day<br>E2: 10 mg, three times a day<br>E2: 2 mg, three times a day	5/20 (25)	9/20 (45)
Iwase et al, 2013	EE: 3 mg	9/18 (50)	10/18 (56)
Chalasani et al, 2014	E2: 6 mg	3/13 (23)	6/13 (46)
Zucchini et al, 2015	E2V: 2 mg/day	0/19 (0)	5/19 (26)

DES, Diethylstilbestrol; EE, Ethinyl estradiol; E2, Estradiol; CR, complete response; PR, partial response; SD, stable disease
